# Peroxisome Proliferator-Activated Receptor and Retinoic X Receptor in Alcoholic Liver Disease

**DOI:** 10.1155/2009/748174

**Published:** 2009-09-14

**Authors:** Tommaso Mello, Simone Polvani, Andrea Galli

**Affiliations:** ^1^Gastroenterology Unit, Department of Clinical Pathophysiology, University of Florence, Viale Pieraccini 6, 50134 Firenze, Italy; ^2^FiorGen Farmacogenomic Foundation, Via Luigi Sacconi 6, 50019 Firenze, Italy

## Abstract

A growing number of new studies demonstrate that nuclear receptors are involved in the development of alcoholic liver disease (ALD). Ethanol metabolism and RXR/PPAR functions are tightly interconnected in the liver. Several ethanol metabolizing enzymes are potently regulated by RXR and PPAR*α* after alcohol consumption. The increased ethanol metabolism, in turn, leads to alteration of the redox balance of the cells and impairment of RXR/PPAR functions by direct and indirect effects of acetaldehyde, resulting in deranged lipid metabolism, oxidative stress, and release of proinflammatory cytokines. The use of animal models played a crucial role in understanding the molecular mechanisms of ALD. In this paper we summarize the reciprocal interactions between ethanol metabolism and RXR/PPAR functions. In conclusion, RXR and PPAR play a central role in the onset and perpetuation of the mechanisms underling all steps of the clinical progression in ALD.

## 1. Introduction

Alcoholic liver disease, which includes a spectrum of liver injury that covers from the relatively benign alcoholic fatty liver to the potentially fatal alcoholic hepatitis and cirrhosis, has a known etiology but a complex pathogenesis resulting from a combination of genetic, environmental, nutritional, metabolic, and more recently, immunologic factors as well as cytokines [1–4]. 

 Fatty liver is the most frequent hepatic abnormality found in alcoholics as a toxic manifestation of ethanol ingestion. Fatty liver may occur alone or be part of the picture of alcoholic hepatitis or cirrhosis and the development of these late alterations is not clearly understood. 

 In the liver, ethanol is metabolized to acetaldehyde by two systems: the cytosolic, largely uninducible, aldehyde dehydrogenase (ADH) and the ethanol inducible microsomal cytochrome P4502E1 [5, 6]. Mitochondrial acetaldehyde dehydrogenase (ALDH) is then responsible for the further oxidation of acetaldehyde to acetate (a nontoxic metabolite) using NAD+ as a substrate.

Ethanol complete degradation produces a large amount of reducing agents in the form of NADH (from ADH and ADLH catalyzed reactions) and NADPH (from cytochrome P4502E1) that overwhelm the hepatocyte's ability to maintain redox homeostasis. Moreover the altered redox state impairs gluconeogenesis, diverts acetyl-CoA toward ketogenesis and fatty acid synthesis, and diminishes lipid oxidation disrupting fatty acids *β*-oxidation [7]. Recent works indicate that reactive oxygen species play a major role in alcohol induced liver injury: CYP2E1 ethanol degradation in the presence of iron generates reactive oxygen species (ROS) thus increasing oxidative stress and leading to membrane-lipids oxidation [8], furthermore oxidative stress depletes the cell reservoir of reduced glutathione (GSH), vitamin E, and S-adenosyl methionine (SAM) [9–12]. These altered mechanisms, along with oxidative stress, are however insufficient to account for all the effects of ethanol consumption and recent works highlight the importance of nuclear receptors and transcription factors in the pathogenesis of liver disease.

 Inflammation is important in the progress of alcoholic liver disease with Kupffer cell being the master regulator (see for recent reviews [1, 2]). In alcoholics and in ethanol-treated animals plasma LPS levels increase; moreover steatosis and ethanol consumption bring to intrahepatic inflammation due to Kupffer cells deregulated release of inflammatory cytokine (especially TNF-*α*). Several possibilities have been proposed to explain the increase of plasma LPS induced by alcohol. Clinical and experimental studies demonstrated a bacterial outgrowth after ethanol administration [13]; however LPS diffuses from intestine at very low levels. The main mechanism involved appears to be an increased intestinal permeability. Numerous studies demonstrated that ethanol disrupts the functional and structural integrity of intestinal epithelial cells resulting in cellular hyperpermeability and gut leakiness [14–16]. Oxidative stress and toxic metabolites accumulation in intestine may explain the increased permeability. Acetaldehyde, produced by ethanol oxidation in intestine, disrupts intestinal epithelial tight junctions and increases paracellular permeability to endotoxins in Caco-2 cell monolayer [17]. Furthermore, ethanol induces, in vitro and in vivo, the overproduction of nitric oxide, mediated by the inducible nitric oxide synthase [18, 19] that causes intestinal barrier dysfunction trough oxidation and nitration of cytoskeletal proteins [20].

 Liver depleted of Kupffer cells shows a decrease in tissue damage induced by ethanol feeding [21]. Kupffer cells produce a large amount of inflammatory citokines, especially TNF-*α*. Kupffer cells from fed-ethanol animals are more susceptible to LPS due to increased expression of Toll-like receptor 4 (TLR-4) [22] that binds LPS and regulates TNF-*α* secretion [23]. Accumulating evidences suggest that ethanol sensitizes Kupffer cells to LPS through the production of reactive oxygen species (ROS) by NAPDH oxydase and CYP2E1 [24].

 Other important factors for the development of alcoholic liver disease whose role became more clear in the last years are some adipokines, such as adiponectin [2] and leptin [25, 26], that are involved in the control of the alcohol induced inflammatory and fibrogenic response.

 Chronic exposure to ethanol inhibits the activity and/or downregulates the expression of several lipid metabolism regulating enzymes, foremost AMP-activated kinase (AMPK) [27], peroxisome proliferator activated receptors (PPARs) [28], retinoid X receptors (RXRs) [29], and sirtuin1 (SIRT1) and 5 (SIRT5) [30, 31], whereas up-regulates the sterol regulatory element-binding protein 1 (SREBP-1) [32]. The mechanisms by which ethanol consumption causes accumulation of hepatic triacylglycerols are complex. AMP-activated protein kinase (AMPK) has a pivotal role in the regulation of lipid metabolism; its activation increases fatty acid oxidation and reduces their synthesis. AMPK activity in liver of ethanol-fed rats is decreased and less sensitive to changes in the AMP/ATP ratio facilitating triacylglycerol accumulation [27]. Activation of SREBP-1 by ethanol feeding is associated with increased expression of hepatic lipogenic genes as well as the accumulation of triglycerides in the livers [32].

 Alcohol significantly reduces SIRT1 and SIRT5 expression [30, 31, 33–35]: cytoplasmic SIRT1 and mitochondrial SIRT5 are (NAD^+^)-dependent deacetylase that regulate the activity of histonic and nonhistonic proteins [36, 37]. They are important regulators of energy metabolism controlling the gluconeogenic genes and hepatic glucose output through PGC-1*α* deacetylation (and hence the gluconeogenesis/glycolitic pathway) [30, 31, 38–40]; in addition, SIRT1 modulates the effects of PGC-1*α* repression of glycolytic genes in response to fasting and pyruvate [39]. Knockdown of SIRT1 in liver causes mild hypoglycemia, increases systemic glucose and insulin sensitivity, and decreases glucose production. SIRT1 knockdown also decreases serum cholesterol and increases hepatic free fatty acids (FFAs) and cholesterol content [40]. Ethanol administration induces PGC-1*α* and p53 hyperacetylation that could be partially ascribed to SIRT1 and SIRT5 reduced expression; posttranslational modifications of these proteins inactivate PGC-1*α* and p53 physiological functions and are associated with mitochondrial dysfunction [30, 31]. Sirtuins may also regulate lipid metabolism: SIRT-1 mediates SBREP-1 activation by ethanol regulating its acetylation. In fact, inhibition of hepatic SIRT1 activity is associated with an increase in the acetylated active nuclear form of SREBP-1c in the livers of ethanol-fed mice [33]. Moreover in ethanol-fed mice, resveratrol, a potent SIRT agonist, prevents alcoholic liver steatosis suppressing SREBP-1 and activating PGC-1a [35].

 PPAR*α* forms heterodimers with RXR to regulate and bind to PPAR response elements (PPREs) of genes involved in the regulation of fatty acid oxidation and transport. Ethanol reduces PPAR*α* activity and RXR protein levels; these reductions are associated to the inhibition of fatty acid oxidation [28, 29].

 A growing number of new studies demonstrate that PPAR and RXR nuclear receptors are involved in many aspects of the development of alcoholic liver disease, ranging from ethanol oxidation to regulation of ethanol-induced inflammatory responses. 

 In this paper we will summarize the progress in the understanding of ethanol metabolism regulation by PPAR and RXR nuclear receptors in alcoholic liver disease.

## 2. Role of PPAR in Alcoholic Liver Disease

In the past decade the role of PPAR receptors in the development of ALD has been intensively investigated, both in cell culture systems and in ethanol-fed rodents. The emerging picture is a complex network of alcohol-induced deregulation of all PPAR isoforms, involving different cell types and mechanisms.

### 2.1. PPAR*α* in Alcoholic Liver Disease

PPAR*α* is a master regulator of lipid metabolism in the liver, controlling the expression of genes involved in the transport, oxidation, and export of free fatty acids. Increased levels of FFA in the hepatocyte activate PPAR*α* by direct binding to the receptor and thus inducing the expression of genes involved in the mitochondrial and peroxisomal FFA *β*-oxidation pathways. In addition to increasing fatty acids disposal through oxidative degradation, PPAR*α* also inhibits the lipogenic pathway by induction of the malonyl Co-A decarboxylase, thus promoting the degradation of malonyl Co-A, a precursor of fatty acid biosynthesis [9]. 

 Since fatty liver represents a very common finding in ALD, the effect of ethanol metabolism on PPAR*α* regulated processes has been intensively investigated in the past ten years.

 In cultured hepatoma cells ethanol affects PPAR*α* transcriptional activity by inhibiting the ability of the receptor to bind its PPRE consensus sequences. This effect is dependent on the ability of the cell to metabolize ethanol to acetaldehyde as it was abolished by the ADH inhibitor 4-methylpirazole and enhanced by the ALDH inhibitor cyanamide. Moreover, administration of acetaldehyde alone to cultured cells inhibited PPAR*α* binding to DNA, strongly suggesting that acetaldehyde is responsible for the effects of ethanol [28].

 In vivo experiments on ethanol-fed rodents reported animal-specific differences on the effects of ethanol on hepatic PPAR*α* protein levels. In Sv/129 mice and in rats, ethanol administration decreased PPAR*α* protein levels [41–43]. Activation of PPAR*α* by clofibrate in ethanol-fed rats ameliorates fatty liver and decreased necroinflammatory injury [43]. On the other hand, in ethanol-fed C57BL/6J mice PPAR*α* protein levels did not change substantially; however PPAR*α*/RXR binding to DNA was significantly impaired and some PPAR*α* target genes (as medium chain acyl CoA dehydrogenase) were downregulated [29]. Although the observed reduction in RXR protein level in ethanol-fed mice could certainly account for the reduced PPAR*α*/RXR DNA binding, induction of PPAR*α* alone by its agonist WY14,643 restored PPAR*α*/RXR binding activity by inducing PPAR*α* but not RXR protein levels, thus revealing that also a reduced activation of PPAR*α* mediates the ethanol effects in vivo. The restored PPAR*α*/RXR binding by WY14,643 administration to ethanol-fed mice was accompanied by an increase of the mRNA of PPAR*α* target genes, some of which were actually either not downregulated by ethanol feeding, such as acyl-CoA oxidase, carnitine palmitoyl transferase-1 (CPT-1), very-long chain acyl CoA dehydrogenase and synthetase, or even induced by ethanol, such as L-fatty acid binding protein (L-FABP) [29]. The induction of PPAR*α* target genes by WY14,643 was accomplished even with concomitant administration of ethanol, indicating that this ligand prevents the effect of ethanol on PPAR*α*, possibly by increasing the fraction of DNA-bound PPAR/RXR and thus minimizing the post-translational modifications of the PPAR*α* DNA binding domain by acetaldehyde [2, 29]. PPAR*α* activation by WY14,643 restored the fatty acid *β*-oxidation, normalized serum fatty acid and trygliceride levels, and prevented fatty liver in ethanol-fed mice.

 Taken together these data suggests that if on one hand ethanol may not completely impair the basal expression of many PPAR*α* target genes, on the other hand it severely reduces the ability of the receptor to induce the lipid oxidative metabolism and detoxification systems, as it is required in response to increased fatty acids intake and alcohol consumption. This line of thought seems to be supported by the experiments conducted in PPAR*α* null mice. In fact in these animals several PPAR*α* regulated genes like the peroxisomal acyl-CoA oxidase, the bifunctional enzyme enoyl-CoA:hydratase-3-3-hydroacyl-CoA dehydrogenase, CYP4A and L-FABP were constitutively expressed at levels comparable to wild-type animals [44, 45].

 However, the induction of both peroxisomal and mitochondrial *β*-oxidation pathways by clofibrate or WY14,643 was completely abolished in PPAR*α*−/− mice [41, 45, 46]. Moreover, PPAR*α* null mice were found to have a marked reduction in the expression of ALDH, which led to increased acetaldehyde levels, increased lipid peroxidation and oxidative stress. PPAR*α* null mice were thus significantly more sensitive to ethanol induced liver damage than wild-type animals [41]. 

 A role for PPAR*α* in hepatic inflammation has also been well established. PPAR*α* null mice have a prolonged inflammatory response through the leukotriene B4 (LTB4) and its receptor compared to wild-type mice [47]. In fact LTB4 is a ligand of PPAR*α*, that acts as an anti-inflammatory receptor stimulating LTB4 degradation in the *β*-oxidative pathways [48]. Moreover, activation of PPAR*α* antagonizes NF-kB signalling, thus preventing the expression of several proinflammatory genes, such as C-reactive protein, fibrinogen –*α* and –*β*, acute-phase response proteins, serum amyloid A [49]. Therefore inhibition of PPAR*α* transcriptional activity by acetaldehyde not only deranges the physiologic lipid metabolism leading to increased ROS and lipid peroxide production, but also stimulates the release of pro-inflammatory cytokines from hepatocytes.

### 2.2. PPAR*γ* in Alcoholic Liver Disease

While ethanol metabolism in the liver occurs largely in hepatocytes, its oxidative product, acetaldehyde, easily diffuses to neighbours cells altering their physiologic processes. In the progression of ALD the activation of Hepatic Stellate Cells (HSCs) is a key step that leads to fibrosis and cirrhosis. Works by our group and others [50–52] demonstrated the importance of PPAR*γ* in the activation of HSC. Active PPAR*γ* is required for the maintenance of the resting “fat storing” phenotype by HSC, and its expression and transcriptional activity decrease during cell activation in culture. Moreover PDGF, a potent inducer of HSC proliferation and migration, induces inactivation of PPAR*γ* by phosphorylation [50, 51]. The decrease in PPAR*γ* transcriptional activity results in an increased synthesis of fibrillary collagens, while activation of the receptor by thiazolidinediones (TZDs) ligands was able to reduce the collagen synthesis in HSC both in vitro and in vivo [53]. The mechanism of PPAR*γ* inhibition in human HSC by ethanol metabolism is rather complex. We found that acetaldehyde inhibits PPAR*γ* by a MAPK mediated phosphorylation on Ser84. The phosphorylation of PPAR*γ* was demonstrated to be dependent on a pathway involving c-Abl, PKC*δ*, and ERK1/2, and to be initiated by acetaldehyde through a H_2_O_2_ dependent mechanism [54]. The signalling axis acetaldehyde-H_2_O_2_-PKC-ERK1/2 in mediating the pro-fibrogenic response of HSC is well established [55–57], and H_2_O_2_ produced in Kupffer cells induces collagen deposition by HSC [58]. Therefore, inhibition of PPAR*γ* by phosphorylation can potentially occur as a consequence of several diverse processes taking place during the ethanol-induced liver injury.

 Inflammation plays a central role in the onset and progression of ALD. PPAR*γ* ligands inhibited the pro-inflammatory behaviour of HSC downregulating the monocyte chemotactic protein-1 (MCP-1) [51]. Pioglitazone prevents liver injury by ethanol and LPS in rats [59, 60]. In this model, activation of PPAR*γ* by pioglitazone was shown to reduce the production of TNF*α* by activated Kupffer cells. Kupffer cells are able to metabolize ethanol [61, 62] and chronic ethanol consumption induces CYP2E1 expression in Kupffer cells [63]. Although in acute alcohol induced-liver injury acetaldehyde has been shown to inhibit TNF-*α* release by Kupffer cells through inhibition of the NF-kB pathway [64, 65], in chronic alcohol abuse the role of Kupffer cell activation and TNF-*α* release in promoting liver injury is well established. It is therefore tempting to speculate that in ALD acetaldehyde, produced in Kupffer cells or hepatocytes, could lead to suppression of PPAR*γ* transcriptional activity and promote the release of TNF-*α*. Indeed, enhanced release of TNF-*α* in response to acetaldehyde or LPS by Kupffer cells isolated from ethanol-fed rats has been reported [66, 67]. Moreover, in ALD increased TNF-*α* release by Kupffer cells does occur by LPS stimulation due to increased intestinal permeability (described elsewhere in this review). Circulating LPS binds to LPS binding protein (LBP) [68] which promotes its interaction with the Kupffer cells' CD14 cell-surface receptor. This complex interacts mainly with the toll-like receptor 4 (TLR4), which in turn transduces the down-stream signal through activation of the PCK, MAPK, and NF-kB signalling pathways [69]. Furthermore, ethanol induces the expression of CD14/TLR-4 receptors in kupffer cells, thus “priming” the liver macrophage population to respond to LPS stimulation, and this sensitization was found to depend upon NADPH oxidase mediated-oxidative stress and activation of the NF-kB pathway [22, 70]. TNF-*α* is also known to downregulate PPAR*γ* function by several mechanisms (see Ye 2008 for a recent review [71]), and LPS treatment was shown to downregulate PPAR*γ* expression in Kupffer cells both in vitro and in an animal model of sepsis through a TNF*α* -dependent mechanism [72]. 

 Finally, a link between CYP2E1 and TNF-*α* production has been described *in vitro* in a macrophage cell line with stable expression of CYP2E1 [24]. In this model, increased expression of CYP2E1 was accompanied by increased levels of CD14/TLR-4, NADPH oxidase, and H_2_O_2_. The higher production of hydrogen peroxide resulted in activation of the MAPKs ERK1/2 and p38, which stimulated TNF-*α* production via activation of NF-kB and stabilization of TNF-*α* mRNA, respectively [24]. 

 Although these interlinked events still need to be substantiate in an animal model of ALD, the emerging picture describes a likely redundant mechanism by which ethanol induction of CYP2E1 and NADPH oxidase systems enhances oxidative stress and sensitizes Kupffer cells to endotoxins, thus promoting inflammation by inhibition of PPAR*γ* function and activation of NF-kB pathways. This line of thought seems to be supported by recent evidences demonstrating that TLR-4 mediates the LPS-induced downregulation of PPAR*γ* by a NF-kB dependent mechanism in macrophages [73]. Loss of PPAR*γ* activity was sufficient to induce a pro-inflammatory state, indicating that PPAR*γ* suppress inflammation under basal conditions by repressing NF-kB activity, while upon activation of TLR4, NF-kB drives down PPAR*γ* expression and thereby obviates any potential anti-inflammatory effects of PPAR*γ* in LPS-stimulated macrophages [73].

### 2.3. PPAR*β*/*δ* and Ethanol

PPAR*β*/*δ* is probably the less characterized isoform of the PPAR family. It is expressed in a large array of tissues, including central nervous system, liver, adipose tissue, muscles, and the gastrointestinal tract. In skeletal and cardiac muscle PPAR*β*/*δ* is expressed at much higher levels than PPAR*α* and PPAR*γ* [74, 75]. Recent studies suggest that, in the adult, PPAR*β*/*δ* plays important roles in maintaining the glucose-lipid homeostasis by stimulating fatty acid oxidation and uncoupling of the respiratory chain in the muscle, by inhibiting glucose and VLDL secretion from the liver, by stimulating the cholesterol efflux increasing circulating HDL levels; moreover PPAR*β*/*δ* is also implicated in the regulation of the inflammatory activity of macrophages (for recent reviews on the metabolic action of PPAR*β*/*δ* see [76, 77]).

 Very little is known about the effect of ethanol on PPAR*β*/*δ*. In rat hepatoma cells, acetaldehyde inhibited PPAR*β*/*δ* DNA binding activity but at a much higher concentration than that required for PPAR*α* inhibition [28]. PPAR*β*/*δ* is also expressed in hepatic stellate cells, and its expression increases with cell activation in vitro and in vivo [78, 79]. However, the role of this PPAR isoform in stellate cells is of difficult interpretation, since if on one hand it promotes hepatic stellate cells proliferation [79], on the other it induces genes involved in the esterification of Vitamin A such as CRBP-1 and LRAT, possibly reflecting a compensatory mechanism aimed to counterbalance the loss of retinol storage during activation [78]. Very recently, the mRNA levels of PPAR*β*/*δ* have been shown to increase in livers of ethanol fed-rats [80]; however in this model activation of PPAR*β*/*δ* by the specific agonist L165,041 resulted in an attenuation of the ethanol-induced hepatic injury and in an improvement of liver regeneration [80].

 Ethanol is known to disrupt insulin signalling [81–83] and to impair liver regeneration [84]. According to recent data demonstrating a role for PPAR*β*/*δ* in the regulation of glucose metabolism and insulin sensitivity [85], the protective effects of PPAR*β*/*δ* agonist on ethanol-induced liver injury were shown to be dependent on the amelioration of the insulin binding to insulin and IGF-1 receptors and activation of the down-stream signalling pathways [80].

 An important role for PPAR*β*/*δ* in the regulation of the inflammatory profile of tissue macrophages has been recently discovered [86, 87]. Odegaard and coworkers showed that PPAR*β* */* *δ* is required for alternative activation of Kupffer cells, and alternatively activated macrophages suppress inflammation and promote tissue repair [87, 88]. Moreover, alternatively activated Kupffer cells promote the *β*-oxidative pathways and suppress lipogenesis in hepatocytes via a paracrine cross-talk [87]. The mouse model used by Odeggard and coworkers was developed to study obesity-induced insulin resistance and type-2 diabetes; however since several parallelisms exist between alcoholic and nonalcoholic liver injury [23], it would be of great interest to test the hypothesis that macrophage specific inhibition of PPAR*β*/*δ* would play an important role in alcohol-induced steatohepatitis.

PPAR*β*/*δ* null mice show defective myelination of the corpus callosum, reduced adipose tissue and altered inflammatory response in the epidermis [89]. In B12 oligodendrocyte-like cells ethanol was shown to selectively reduce PPAR*β*/*δ* expression by increased mRNA degradation without affecting PPAR*α* and PPAR*γ* [90]. These observations could possibly undercover a mechanism underling the ethanol-induced myelination defects and neurological impairment in the foetus [91]. In a recent study by Venkata et al. PPAR*α* and *β*/*δ* were shown to be differentially affected by acetaldehyde and ethanol, respectively. The authors show that in MCF-7 breast cancer cell line acetaldehyde inhibits PPAR*α* but not PPAR*β*/*δ*, while the latter is inhibited directly by ethanol [92]. A growing body of evidence suggests an association between ethanol consumption and increased cancer risk in different tissues, including breast [93, 94]. More studies are needed to elucidate the effects of ethanol metabolism on PPAR*β*/*δ* and the underling mechanisms, but it is intriguing to speculate that some effects of ethanol on peripheral tissues could be mediated by this poorly understood PPAR isoform.

## 3. RXRs, Ethanol Metabolism, and Alcoholic Liver Disease

RXR is a nuclear receptor expressed in almost every cellular type and tissue. Three isoforms of RXR have been found in human, named RXR*α*, *β*, and *γ*, being the *α*-isoform the most abundant in the liver [95]. Unique among the other nuclear receptors, RXR*α* play a major modulatory role across multiple cellular pathways forming mandatory heterodimers with other nuclear receptors.

 RXR *β* and *γ* apparently play minor roles in the liver: it has been demonstrated in mice that RXR*α* null/RXR*γ* null and RXR*β* null/RXR*γ* null mutant phenotypes were indistinguishable from those of RXR*α* null and RXR*β* null mutants, and that the presence of a single allele of RXR*α* is sufficient to perform most of RXR functions [96].

 In the last years there has been an increasing interest in the role of RXR, especially RXR*α* in alcoholic liver disease. Accumulating evidence suggests that RXR may play a major role in many aspects of ethanol metabolism being its expression downregulated by ethanol [42, 97]. In fact, mice fed with ethanol show reduced ability of PPAR/RXR heterodimers to bind DNA and reduced RXR expression [29]. These effects are acetaldehyde dependent because blocking ADH reduces, while blocking ADLH increases, the observed phenotype [28]. Moreover, the human aldehyde dehydrogenase-2 promoter contains a retinoid response element (designated FP330-3′), which may contribute to the regulation of the gene. Heterodimers of retinoic acid receptor (RAR)*α*, *β*, and *γ* with RXR*α* bound the FP330-3′ site, stimulating the expression of reporter constructs containing the FP330-3′ sites in a 9-*cis* retinoic acid-dependent fashion in cultured cells.

 More insights on RXR specific role in alcoholic liver disease come from experiments with animal models mainly from the group of Professor Wan JY that demonstrated an altered ethanol methabolism in RXR*α* null mice.

 Hepatocyte RXR*α*-deficient mice possess a phenotype partially overlapping both PPAR*α* and PPAR*γ* knockout-mice phenotypes. Like the PPAR*α*-null mice, hepatocyte RXR*α*-deficient mice are obese, have a larger fat mass, and higher serum cholesterol and leptin levels compared with wild-type mice; similar to PPAR*γ* +/− mice, hepatocyte-RXR*α*-deficient mice also have reduced food intake and increased serum leptin levels [98]. On the contrary, hepatocyte RXR*α* deficiency results in an improved glucose tolerance without altering insulin level or insulin sensitivity [98].

 RXR*α* deletion increases liver enzymatic activity of ADH1 isoform without affecting ADH2 and ADH3 [12]. It has been suggested that RXR*α* may regulate ADH1 translation because mRNA levels were the same between null and wild-type mice, while there is increased abundance of ADH1 mRNA in the polysome fraction, indicating higher translation in null mice. This latter effect may be mediated by leptin whose levels are increased in RXR null mice [98] and has been reported capable of regulating ADH activity [99].

 RXR may regulate acetaldehyde oxidation to acetate and its clearance. ALDH mRNAs (especially ALDH1 mRNA) are reduced in null mice indicating a possible direct regulation that would parallel with the above mentioned finding that human ALDH promoter responds to RXR. Increased ADH1 activity accounts for the rapid oxidation of ethanol to acetaldehyde; reduced ALDH activity observed in RXR deficient mice impairs the further oxidation to acetate sensitizing the mice to the ethanol-induced damage. 

 RXR is able to regulate the expression of many xenochemical metabolizing enzymes of the cytochrome P450 family acting as a dimerization agent for PPAR, LXR, PXR [100–103], but its ability to regulate the cytochrome P450 CYP2E1, involved in ethanol oxidation to acetaldehyde, needs further investigation. The promoter region of cytochrome P4504A contains an imperfect direct repeat sequence recognized by PPAR*α*/RXR heterodimers and P4504A is induced by peroxisome proliferators [104] whereas CYP2B is induced by androstane receptor/RXR heterodimers [105]. It has been demonstrated that ethanol-fed animals show a reduced RXR expression whereas CYP2E1 mRNA increases [42, 97]. In an RXR null background, male mice but not female have a reduced expression of CYP4A, 3A, CYP2A and CYP2B mRNA [10, 106] but not of CYP2E1 [106] compared to male wild-type mice. Furthermore, when RXR null mice were challenged with ethanol, induction of CYPs expression was lower in mutant mice compared to wild-type mice [10]. In RXR KO mice CYP2E1 activity is reduced [12] and the enzyme is not induced by ethanol [107]. The apparent discrepancy between wild-type and RXR KO mice in the activation of cytochrome CYP2E1 may be due to residual RXR expression in ethanol-fed animals compared to RXR KO mice where RXR*α* has been knocked out. It also may suggest that RXR may play an important role in P450 regulation at the transcription and translational levels.

 The altered expression of cytochrome enzymes in RXR KO mice suggests that these mice could be less sensitive to ethanol damage with respect to wild-type mice; however, ethanol treated KO mice still show induced liver damage. Toxic acetaldehyde accumulation due to altered ADL/ADLH activities (as described above) may account for that damage but other mechanisms are also involved.

 It is well known that ethanol alters phase II metabolism of xenobiotics agents [108]. Depletion of GSH reservoirs reduces the liver ability to withstand oxidative stress from altered redox equilibrium and to prevent lipids peroxidation as a result of ethanol ingestion. Dai et al. [10] and Gyamfi and Wan [109] demonstrated that GSH levels are reduced in wild-type mice after ethanol administration; this effect is exacerbated in RXR null mice [10, 109]. Compared to wild-type mice, hepatocyte RXR*α*-deficient mice have significant lower levels of SAM (a precursor for GSH synthesis) and GSH, which is further reduced after alcohol treatment [10, 11]. The Glutathione S-transferase (GSTs) is a multigene family of enzymes that bind GSH to xenobiotics agents facilitating theirs dissolution in the aqueous cellular and extracellular media, and, from there, out of the body. 

 GSTs are encoded by at least 9 gene family: eight of them encode for cytosolic enzymes (namely, alpha, mu, theta, pi, zeta, sigma, kappa and chi—also called omega) whereas a ninth family, composed at least by six genes encode for microsomal enzymes [110]. GST enzymes of alpha, mu, and pi families account for the majority of cytosolic GST activity.

 In RXR null mice GST activity and transcription is reduced compared to wild-type mice [11, 12]. In hepatocytes, RXR*α* deficiency changes the gene expression profiles of the GSTs: it has been reported that basal expression of 13 out of 15 GST genes was altered in hepatocyte-specific RXR*α*-deficient mice [11], being either down or upregulated. The enzymatic activity of both mitochondrial and cytosolic GSTs is reduced in null mice [12]. Acute ethanol exposure of primary mouse hepatocytes reduces GSH levels and cytosolic GST enzymes activity along with the release of GST into the culture medium. Specific substrates for the mu and pi class demonstrate that ethanol significantly decreases the mu and pi class GST activity by 53% and 13%, respectively. These biochemical changes elicited by ethanol were also accompanied by increased lipid peroxidation and a decreased SAMe to SAH ratio, early biochemical features of ethanol toxicity. Moreover, hepatocytes isolated from RXR KO mice show a decrease of mu and pi class GST activity compared to wild-type mice.

 In H4IIE hepatocytes GSTA2 gene is induced by PPAR*γ* activator and 9-*cis*-retinoic acid. The TZD PPAR*γ* agonists, troglitazone, rosiglitazone, and pioglitazone, in combination with retinoic acid, increase GSTA2 induction, confirming that the activation of PPAR*γ* */*RXR heterodimer contributes to GSTA2 expression [111]. Unique among the GSTs, the alpha class isoenzymes have selenium independent antiperoxidase activity [112]. It has been demonstrated that alpha class GST [113–115] plays an important role in the protection mechanisms against oxidative stress induced by lipid peroxidation.

 This latter finding suggests that in alcoholic liver disease and ethanol metabolism, RXR regulation of GSTs expression may be important not only for conjugation of toxic metabolites to GSH but also for the protection against the deleterious effects of lipid peroxidation.

 Deranged lipid metabolism and steatosis is one of the hallmark of heavy ethanol consumption. The role of nuclear receptors (especially PPAR*α*) in the control of lipid metabolism is well known and has been already extensively reviewed [1, 2, 116, 117]; however, some specific effects, observed in ethanol fed-animals and previously ascribed to the PPAR*α* pathway, could indeed be RXR specific [98, 118]. When fastened or treated with WY14,643, RXR null mice show a phenotype in several aspects different from PPAR null mice. Apolipoprotein A-I and C-III mRNA levels, serum cholesterol and triglyceride levels are markedly induced in untreated RXR null but not in PPAR*α*-null mice. Moreover, fasting-induced PPAR*α* activation and WY14,643 effects are reduced in RXR KO mice. Acyl-CoA oxidase, medium chain acyl-CoA dehydrogenase, and malic enzyme induction by WY14,643 are reduced in null mice without altering their mRNA levels [118].

 In RXR null mice L-FABP levels are dramatically reduced compared to wild-type animals [107]. L-FABP is a PPAR*α* target gene and is necessary for the transport of fatty acids. Ethanol feeding increases hepatic FFA levels both in wild-type and null mice; however in RXR null mice L-FABP is not induced by ethanol and this effect is associated with accumulation of FFA and ethanol induced liver damage [107]. A recent paper from Razny et al. further demonstrated the RXR specific role on lipid metabolism and angiogenesis. Microarray studies on RXR*α* deficient mice fed with a high-fat diet for 7 weeks demonstrated a down-regulation of genes related to angiogenesis, whereas genes involved in adipogenesis, apoptosis, and inflammation were upregulated. Based on these results Razny et al. suggested that impaired fatty acid metabolism in liver leads to impaired angiogenesis due to lipotoxicity and promotion of adipogenesis [119].

 Liver damage resulting from chronic ethanol consumption is also caused by inflammatory processes. As previously reported, in alcoholics increased LPS levels and altered metabolism induce inflammation, worsening alcoholic liver disease, and Kupffer cells are more sensitive to LPS due to increased expression of Toll-like receptor 4 [23]. In the negative acute hepatic phase response, LPS induces a downregulation of lipid metabolism associated with increased serum triglyceride levels and reduced lipid *β*-oxidation. These effects appear to be mediated by a reduction of nuclear receptors expression mediated by TNF-*α* and IL-1 but not IL-6 [120]. RXR*α* expression is lower in LPS negative acute hepatic phase response [101] and HepG2 IL-1 *β*-cells showed a marked decline in RAR/RXR binding to the Ntcp gene that regulates bile flow [121]. The alteration of RXR pathways is associated with changes in its subcellular localization characterized by increased cytoplasmic and reduced nuclear levels [122]. The nuclear residence of RXR*α* is maintained inhibiting c-jun N-terminal kinase or CRM-1-mediated nuclear export, while IL-1 increases the proteasome mediated RXR degradation [123]. 

 Interestingly, hepatocytes from ethanol-fed RXR null mice show an increased NF-kB activation and a strong and higher induction of inflammatory cytokines TNF-*α*, IL-1 and IL-6, compared to wild-type mice [107]. Apoptosis in RXR null mice increases after ethanol ingestion due to a lower expression of antiapoptotic proteins Bcl2 and Bcl-XL, even in the presence of higher levels of IL-6. RXR seems to influence IL-6/STAT3 mediated signalling circumventing STAT3 phosphorylation [107]. This suggests that RXR is an important regulator of inflammatory processes in response to ethanol; it may also be speculated that some of the effects in response to LPS (as describer above) may be ascribed to RXR reduced expression.

 Finally, ethanol and retinoid metabolisms are widely interconnected [124]. Liver from alcoholics shows a marked depletion of Vitamin A that correlates with the activation of stellate cells, their loss of lipid droplets and differentiation in myofibroblast-like cells. The role of nuclear receptors in stellate cells activation is widely studied and it is reported elsewhere in this paper. Key enzymes in ethanol metabolism may act also on retinol pathway [125]. Enhanced activities of ADH, ALDH, and CYP2E1 in alcoholics may account for the observed reduction of Vitamin A in liver cells. Even if retinol it is not their supposed primary substrate it is well known that ADHs and ALDHs may catalyze the conversion of Vitamin A to retinoic acid accelerating retinol metabolism; furthermore alcohol induction of cytochrome CYP2E1 determines a higher rate of Vitamin A catabolism in polar metabolites in the liver. Ethanol may also increase retinol mobilization from liver increasing hydrolization of retinyl esters [126–128]. Alcohol-induced effects on retinol-regulated genes are further increased by RXR downregulation observed in alcohol-fed animals. This establishes a complex regulatory mechanism between RXR, retinol metabolism, and ethanol that is deregulated during heavy ethanol consumption.

## 4. Summary and Conclusions

Ethanol metabolism and RXR/PPAR functions are tightly interconnected in the liver. Several ethanol metabolizing enzymes are potently regulated by RXR and PPAR*α* after alcohol consumption. The increased ethanol metabolism, in turn, lead to alteration of the redox balance of the cell and impairment of RXR/PPAR functions by direct and indirect effects of acetaldehyde, resulting in deranged lipid metabolism, oxidative stress, and release of pro-inflammatory cytokines. In this paper we summarized the reciprocal interaction between ethanol metabolism and RXR/PPAR functions.

 The use of alcohol-fed rodents played a crucial role in understanding the molecular mechanisms of ALD. However, important differences exist in the regulation of the oxidative metabolism between rodents and humans. In fact, while in rodents the need for increased oxidative metabolism induces peroxisome proliferation, humans exclusively respond by increased mitochondrial *β*-oxidation. Moreover, the levels of PPAR*α* receptor are markedly lower in humans than in rodents, and this may be even more striking during alcohol abuse. PPAR and RXR KO mouse models highlighted the reciprocal regulation of PPAR, RXR and ethanol in alcoholic liver disease. In conclusion, RXR, and PPAR play a central role in the onset and perpetuation of the mechanisms underlying all steps of the clinical progression in alcoholic liver disease.
